# Effektivität einer sequenziellen versus einer simultanen Radiochemotherapie oder alleinigen Radiotherapie bei der adjuvanten Behandlung des Zervixkarzinoms nach Hysterektomie

**DOI:** 10.1007/s00066-021-01796-z

**Published:** 2021-05-30

**Authors:** Kilian Schiller, Stephanie E. Combs

**Affiliations:** 1 Klinik und Poliklinik für RadioOnkologie und Strahlentherapie, TU München, München, Deutschland; 2 Institut für Innovative Radiotherapie (iRT), Department of Radiation Sciences (DRS), Helmholtz Zentrum München (HMGU), München, Deutschland; 3 Deutsches Konsortium für Translationale Krebsforschung (DKTK), Partner Site Munich, München, Deutschland

## Ziel der Arbeit

Das Ziel der Arbeit ist der postoperative Vergleich einer sequenziellen Radiochemotherapie mit einer simultanen Radiochemotherapie sowie einer alleinigen Radiotherapie bei Patientinnen mit Zervixkarzinom in frühen Stadien.

## Patientengut und Methoden

In diese Studie wurden 1048 Patientinnen aus acht chinesischen Kliniken eingeschlossen und nach Hysterektomie und Lymphadenektomie randomisiert auf die drei Therapiearme:

- Alleinige Strahlentherapie (RT)
- Simultane Radiochemotherapie (CCRT) mit wöchentlichem Cisplatin 30–40 mg/m^2,^
- Sequenzielle Radiochemotherapie (SCRT) mit Cisplatin 60–65 mg/m^2^ + Paclitaxel 135–175 mg/m^2^ q21 – 2 Zyklen Chemotherapie jeweils vor und nach der Strahlentherapie

Einschlusskriterien waren ein FIGO-Stadium IB bis IIA *mit mindestens einem *Risikofaktor (Lymphknotenmetastase[n], Parametrienbefall, Resektionsränder nicht frei, Lymphangiose oder tiefer Stromabefall). Für die Strahlentherapie wurde eine Gesamtdosis von 45 bis 50 Gy angestrebt. Eine Stratifizierung erfolgte nach der Tumorgröße (4 cm vs. > 4 cm). Der primäre Endpunkt war das krankheitsfreie Überleben (DFS). Sekundäre Endpunkte umfassten das Gesamtüberleben (OS), das nichtlokale DFS und die Toxizität.

## Ergebnisse

Die Therapiearme waren ausgewogen zusammengesetzt bezüglich Risikofaktoren (bis auf die Rate an Lymphknotenbefall), RT: 18,3 % vs. CCRT: 30,1 % vs. SCRT: 29,7 %. Die Mehrheit der Patientinnen wurde mit einer offenen Resektion behandelt. 7,7 % wurden operativ laparoskopisch versorgt. Bei der Gruppe mit großen Tumoren > 4 cm erhielten 84,4 % der Patientinnen eine neoadjuvante Chemotherapie. Eine vollständige Komplettierung nach Protokoll war in 98,8 %, 62,3 % und 73,4 % der Fälle möglich für die Gruppen RT, CCRT und SCRT. Das mittlere Zeitintervall von der Operation bis zur Einleitung der Therapie betrug in der RT-Gruppe 32 Tage, in der CCRT-Gruppe 33 Tage und in der SCRT-Gruppe 8 Tage.

Nach einer mittleren Nachbeobachtungszeit von 56 Monaten erlitten 16,4 % der Patientinnen ein Rezidiv oder verstarben tumorbedingt.

Die SCRT war assoziiert mit einer höheren Wahrscheinlichkeit für ein DFS nach drei Jahren im Vergleich zur RT (90 % vs. 82 %, HR 0,61 [95 %-KI 0,42–0,89], *p* = 0,01) und CCRT (90 % vs. 85 %, HR 0,67 [95 %-KI 0,46–0,99], *p* = 0,04). Dieser Unterschied war noch bedeutender nach Adaptierung an den Lymphknotenstatus. Außerdem wies die Gruppe SCRT weniger Fernmetastasen nach drei Jahren auf (6,5 %) im Vergleich zu RT (10,6 %) und CCRT (11 %). Des Weiteren senkte die SCRT die krankheitsspezifische Todesrate im Vergleich zur alleinigen RT (HR 0,58, *p* = 0,03).

Die Unterschiede im DFS und OS waren nicht signifikant zwischen der RT- und CCRT-Gruppe.

In der Subgruppenanalyse konnte sich die SCRT im Vergleich zur RT immer signifikant absetzen bezüglich der Hazard Ratio, außer bei Tumoren > 4 cm, die in den meisten Fällen eine neoadjuvante Chemotherapie erhalten hatten. Anders die CCRT, die nur in der Subgruppe der Karzinome mit zumindest adenomatöser Komponente einen signifikanten Vorteil zur alleinigen RT aufwies.

Therapieassoziierte Todesfälle traten nicht auf. Grad-III- oder Grad-IV-Nebenwirkungen waren in der RT-Gruppe seltener (12,9 %) als in der CCRT- (28,5 %) und SCRT-Gruppe (25,3 %).

## Schlussfolgerung der Autoren

Da die SCRT im Vergleich zur CCRT und RT das DFS verbessert und sowohl die Fernmetastasierung als auch das krankheitsspezifische Todesrisiko senkt, sollte sie als bevorzugtes Therapieschema gelten.

## Kommentar

In der postoperativen Situation sind beim Zervixkarzinom Risikofaktoren definiert, die auch als „Sedlis-Kriterien“ und „Peters-Kriterien“ nach den beschreibenden Autoren bekannt sind [1, 2]. Bei Vorliegen mindestens eines der Peters-Kriterien (pN+, Parametrienbefall, R1) ist eine Hochrisikosituation gegeben. Über die Sedlis-Kriterien (Lymphangiose, Tumor > 4 cm, > 1/3 Stromainvasion) wird ein mittleres Risiko angenommen. Als Einschlusskriterium in der vorliegenden Studie war mindestens eines dieser Kriterien zu erfüllen, ausgenommen die Tumorgröße, die nicht ausreichend gesichert war.

Die Vorteile, gemessen am progressionsfreien Überleben und Gesamtüberleben der kombinierten Radiochemotherapie, wurden für Hochrisikopatientinnen in der GOG-109-Studie gezeigt [2, 3]. Das begleitende Chemotherapieregime (Cisplatin + 5-FU) unterschied sich allerdings hier von dem der hier besprochenen Studie. Eine Subgruppenanalyse konnte wegen zu kleiner Fallzahlen in dieser Studie keinen Vorteil mehr in einer der Therapiegruppen aufzeigen. Dennoch ist die RCT seitdem in der postoperativen adjuvanten Situation für Patientinnen mit mittlerem und hohem Risiko gesetzt.

Dies wirft die Frage auf, warum die CCRT in der Arbeit von Huang und Kollegen nicht besser abschnitt als die alleinige RT. Womöglich liegt es allein an der reduzierten Chemotherapie als Monotherapie mit Cisplatin.

Das deutlich bessere Abschneiden der SCRT im Vergleich zur CCRT ist vermutlich nicht nur durch die aggressivere Chemotherapie bedingt. Auch die zeitliche Komponente mit dem zeitnahen Beginn der SCRT nach der Operation scheint als begründender Aspekt logisch.

Huang et al. entschieden sich im SCRT-Arm für ein geändertes Therapieschema mit Platin und einem Taxan und begründeten dies mit guten Effekten bei akzeptablen Nebenwirkungen in fortgeschrittenen Fällen in vorausgegangenen Studien [4, 5]. Konsequenterweise war der Effekt des Vorteils der SCRT in der Subgruppe der Hochrisikopatientinnen besonders ausgeprägt. Dies spiegelt sich auch in der erniedrigten Rate an Fernmetastasen wider, was dafür spricht, dass in diesem Setting die adjuvante Therapie, zumindest für einen Teil der Patientinnen, nicht nur das Lokalgeschehen kontrolliert, sondern auch eine Metastasierung in andere Organsysteme. In der vorliegenden Analyse profitierten alle Patientinnen von dem aggressiveren Schema, im Vergleich zur RT allein, bezüglich der onkologischen Endpunkte.

Die Ergebnisse der GOG-263-Studie könnten noch weitere Erkenntnisse liefern zur Frage, welche Patientinnen wohl besonders von einer Eskalation der Chemotherapie profitieren könnten (NCT01101451), besonders im Hinblick auf den Lymphknotenstatus.

## Fazit

Mit der hier diskutierten Studie positioniert sich die sequenzielle Radiochemotherapie als vielversprechende Möglichkeit. Für sie sprechen der zeitnahe postoperative Beginn und die geringeren Nebenwirkungen im Vergleich zur simultanen Radiochemotherapie. Einen Vergleich mit einem aggressiveren Behandlungsschema als CCRT (keine Monotherapie) lässt die aktuelle Studie nicht zu.

*Kilian Schiller und Stephanie E. Combs, München*
